# Boric acid impedes glioblastoma growth in a rat model: insights from multi-approach analysis

**DOI:** 10.1007/s12032-025-02600-z

**Published:** 2025-01-17

**Authors:** Hasan Turkez, Fatih Alper, Cemil Bayram, Cem Baba, Edanur Yıldız, Melik Saracoglu, Muhammed Kucuk, Berrah Gozegir, Metin Kiliclioglu, Mustafa Yeşilyurt, Ozlem Ozdemir Tozlu, Ismail Bolat, Serkan Yildirim, Muhammed Furkan Barutcigil, Fatih Isik, Özlem Kiki, Fahri Aydın, Mehmet Enes Arslan, Kenan Cadircı, Adem Karaman, Abdulgani Tatar, Ahmet Hacımüftüoğlu

**Affiliations:** 1 Department of Medical Biology, Faculty of Medicine, Atatürk University, Erzurum, Turkey; 2 Department of Radiology, Faculty of Medicine, Atatürk University, Erzurum, Turkey; 3 Department of Pharmacology and Toxicology, Faculty of Veterinary Medicine, Atatürk University, Erzurum, Turkey; 4 Department of Molecular Biology and Genetics, Faculty of Science, Erzurum Technical University, Erzurum, Turkey; 5 Trustlife Labs, Drug Research & Development Center, Istanbul, Turkey; 6 Department of Pathology, Veterinary Faculty, Atatürk University, Erzurum, Turkey; 7 Department of Medical Biochemistry, Faculty of Medicine, Atatürk University, Erzurum, Turkey; 8 Department of Internal Medicine, Erzurum Regional Training and Research Hospital, Health Sciences University, Erzurum, Turkey; 9 Department of Medical Genetics, Faculty of Medicine, Atatürk University, Erzurum, Turkey; 10 Department of Medical Pharmacology, Medical Faculty, Atatürk University, Erzurum, Turkey

**Keywords:** Anticancer, Boric acid, Boron, Glioblastoma, Xenograft rat model

## Abstract

Limited advancements in managing malignant brain tumors have resulted in poor prognoses for glioblastoma (GBM) patients. Standard treatment involves surgery, radiotherapy, and chemotherapy, which lack specificity and damage healthy brain tissue. Boron-containing compounds, such as boric acid (BA), exhibit diverse biological effects, including anticancer properties. This study aimed to examine whether boron supplementation, as BA, can inhibit glioblastoma growth in a xenograft animal model. Using MRI-based tumor size measurement, survival rates, hematological, clinical biochemistry analyses, and genotoxicity parameters, we assessed the impact of BA. Histopathological, immunohistochemical, and immunofluorescence examinations were also conducted. All BA doses (3.25, 6.5, and 13 mg kg^−1^ b.w.) extended survival compared to GBM controls after 14 days, with a dose-dependent anti-GBM effect observed in MRI analyses. BA treatment improved hematological (WBC and PLT counts) and biochemical parameters (LDL-C, CREA, and ALP). Histopathological examination revealed a significant reduction in tumor diameter with 6.5 and 13 mg kg^−1^ BA. Immunohistochemical and immunofluorescence staining showed modulation of intracytoplasmic Ki67, cytoplasmic CMPK2, and GFAP expressions in tumor cells post-BA treatment. Additionally, BA did not increase micronuclei formations, indicating its non-genotoxic nature. In conclusion, targeting tumor suppressor networks with boron demonstrates significant therapeutic potential for GBM treatment.

## Introduction

Glioblastoma (GBM), categorized as a grade IV astrocytoma, is a highly aggressive form of brain tumor characterized by a diverse array of genetically unstable cells that display resistance to chemotherapy. Typically, surgery alone proves inadequate for GBM treatment due to the challenging nature of achieving complete tumor removal without causing harm to surrounding healthy brain tissue [1]. The standard treatment protocol for newly diagnosed GBM patients typically involves a minimally invasive surgical procedure, followed by radiotherapy combined with concurrent chemotherapy using agents such as temozolomide (TMZ) and carmustine (BCNU). Nevertheless, the current regimen exerts a lack of specificity towards tumor cells and have the adverse effect on healthy brain tissue, resulting in unsatisfactory treatment outcomes [2, 3]. Despite the utilization of modern therapies, GBM remains to be fatal, with poor prognosis and a median survival of only 14 months [4]. In recent years, there has been growing interest in the anti-GBM potential of repurposed drugs, driven by the limited availability of targeted treatments for GBM [5].

There are several limitations that make GBM treatment difficult. Within the context of treatment of GBM, the presence of intratumor heterogeneity represents a notable impediment to attaining positive treatment outcomes. The restricted distribution of drugs within the brain, arising from the intrinsic barrier of the blood–brain barrier (BBB), presents a crucial obstacle in achieving successful drug delivery for GBM treatment [6]. Again, even with the moderate improvements in patient survival seen with certain chemotherapeutics such as TMZ and BCNU, the development of resistance to these drugs remains a substantial barrier to exerting positive clinical outcomes [7]. Indeed, despite the emergence of innovative therapies, several obstacles undermine the motivation of drug developers to continue these efforts. These challenges include the exorbitant cost, high failure rate, and relatively small patient population [8].

Boron is typically regarded as an essential trace element for several organisms involving bacteria and plants. Boron has not been classified as essential for humans as of yet due to limited biochemical proofs but recent studies suggest its necessity for animals and humans [9]. Boron compounds serve various purposes, finding use in glass, nuclear waste storage, alloys and metals, space exploration, fertilizers, insecticides, food supplements, cosmetics, pharmaceuticals as well as personal care items.

Boron and its compounds have attracted significant interest in the field of medicinal chemistry due to their distinctive chemical properties, particularly their electrophilic nature. This characteristic enables boron-containing compounds (BCCs) to engage with diverse biological targets, making them highly valuable in the development of innovative therapeutics. From a chemistry perspective, boron is a highly versatile atom. As a Lewis acid, it features an empty p-orbital, enabling it to act as an electrophile. Numerous studies highlighted here demonstrate boron’s potential to function as a warhead, forming covalent bonds with its targets. This capability is particularly significant in the development of anti-infective and anticancer agents [10]. Due to boron’s expanding role in medicinal chemistry, an increasing number of BCCs are being explored as potential drug candidates [11]. Notably, compounds like boric acid (BA) and sodium tetraborate (borax, BX) have shown beneficial effects on human health. Reported biological benefits by several boron derivatives include antibacterial [12], antiviral [13], antioxidative [14], anti-inflammatory [15], neuroprotective [16], anti-genotoxic [17], and metal chelating [18] properties. Given these well-established biological effects by boron-containing compounds (BCCs), novel boron-based hybrids are considered as promising structural platforms for developing innovative drugs to manage both acute and chronic diseases, rare diseases, as well as cancers.

Boron’s therapeutic efficacy in cancer treatment is believed to be influenced by its position in the periodic table. Due to its electronic similarity to carbon, boron serves as an excellent bioisosteric substitute, offering the potential to enhance drug potency while minimizing toxicity [19]. Hence, boron has become a pivotal asset in anticancer research, primarily due to its significant influence on cell proliferation. In fact, different BCCs have proven effective as inhibitors that target a diverse array of cancer-related proteins, including proteasome, arginase, hypoxia-inducible factor-1α, steroidal sulfatase, and prostate-specific antigen [19]. In line with this background, we previously investigated the anticancer potential by BA and BX on human U-87MG cells and revealed both BA and BX induced cell death via altering cellular proliferation rates, oxidative status, and proinflammatory cytokines. It was noteworthy that BA exerted higher potency against GBM cells compared to BX [20]. Interestingly, recent studies executed that BA could be utilized as a chemo-sensitizer agent to potentially overcome cancer drug resistance in GBM [21] and relatively low doses of BA demonstrated a stabilizing effect by regulating the permeability of the BBB through the inhibition of proteases [22]. Recent studies have suggested that environments rich in boron may have played a beneficial role in the formation of nucleotides [23]. Hence, antitumoral effects by boron may be linked to its inhibition of cytidine/uridine monophosphate kinase 2 (CMPK2), a key enzyme in nucleotide biosynthesis. CMPK2 catalyzes the conversion of CMP and UMP to their diphosphates, essential for DNA and RNA synthesis, making it crucial for cancer cell proliferation and survival [24, 25]. In addition, CMPK2 plays a role in immune response regulation and mitochondrial DNA synthesis, influencing immune pathways and oxidative stress, which are vital for GBM progression [26]. Targeting CMPK2 could disrupt tumor proliferation and modulate the immune microenvironment, offering a multifaceted therapeutic approach. Given its role in metabolic pathways, redox balance, and mitochondrial function, CMPK2 presents an attractive target for GBM treatment [27–29]. In this study, we aimed to evaluate the potential of boron supplementation (as BA) in inhibiting glioblastoma growth in a xenograft animal model, specifically assessing its role as a CMPK2 inhibitor, and contributing to the emerging interest in CMPK2 as a therapeutic target in GBM. Using MRI-based tumor size measurements, survival rates, hematological and clinical biochemistry assessments, genotoxicity analysis, and histopathological, immunohistochemical, and immunofluorescence examinations, we assessed the in vivo anti-glioblastoma effects of BA. This proof-of-concept study explores whether boron compounds could serve as natural sources for developing novel therapeutics for the prevention, treatment, and management of glioblastoma.

## Material and methods

### Chemicals

BA (H_3_BO_3_, CAS No. 10043-35-3, 61.83 g·mol^−1^, ≥ 99.9% pure) was purchased from Sigma-Aldrich (St. Louis, MO, USA). All additional chemicals and reagents were of the analytical reagent grade and were obtained from commercial sources. BA was dissolved in saline solution to prepare different concentrations for the experiments.

### Cell cultures

U87-MG human cells (ATCC® HTB¬14™) were kindly provided by Dr Ahmet Hacımüftüoglu and cultivated in DMEM medium (Thermo Fisher Scientific, Waltham, MA) with 15% fetal bovine serum and 1% penicillin–streptomycin. The cell cultures were kept at 37 °C in a 5% CO_2_ incubator [30].

### Cell implantation and GBM rat model

Every conceivable effort was made to minimize suffering and alleviate potential stress for the animals, and all procedures were conducted in strict adherence to Directive 2010/63/EU of the European Parliament and of the Council, dated September 22, 2010, which outlines regulations for the protection of animals used in scientific research. This study has received approval from the Ethics Committee of Laboratory Animals (HADYEK) at Atatürk University (Approval number: E-16423269-604.02.01/938; Date: 14.05.2023; Decision Number: 05/109). Female Sprague-Dawley rats aged 6–8 weeks and weighing between 260 and 320 g, obtained from ATADEM (Erzurum, Turkey), were utilized for this study. The animals were housed in group settings under standard conditions. They had unrestricted access to water from the site’s drinking water supply and were provided with a standard rodent diet from Bayramoglu Feed Factory, located in Erzurum, Turkey.

The rats were anesthetized using 5% isoflurane, then maintained at 2% isoflurane with a 1 L min^−1^ oxygen flow rate, and positioned in a stereotactic frame (Stoelting Co., IL) for cell implantation. The fur on the top of their heads was shaved to expose the skin, and a mark was made at the bregma following an incision. A burr hole was drilled 1 mm anterior and 3 mm lateral to the right bregma. Using a microsyringe (Hamilton 1700 series), 5 × 10^4^ U87-MG cells suspended in 5 μL of medium at 37 °C were injected to a depth of 4 mm into the brain at a rate of 3 μL min^−1^. Bone wax was applied to seal the burr hole and prevent reflux of the cell suspension. The incision was sutured, and 1 mL of meloxicam (5 mg mL^−1^; Metacam, Boehringer Ingelheim Vetmedica, Ingelheim, Germany) was administered via subcutaneous injection [31, 32].

### Treatment of animals

Fourteen days post-inoculation, tumor development was confirmed through magnetic resonance imaging (MRI). Rats with confirmed tumor growth were randomly assigned to four different treatment groups (*n* = 12) as follows: (1) untreated (GBM group); (2) treated with 3.25 mg/kg BA (B1 group); (3) treated with 6.5 mg kg^−1^ BA (B2 group); (4) treated with 13 mg kg^−1^ BA (B3 group). The animals that were not inoculated tumor cells serve as healthy groups of rats (*n* = 8). Treatments with the BA were administered orally by gavage for 14 consecutive days. The BA doses were selected based on findings from previous studies [33, 34]. Specifically, these doses were chosen because they demonstrated optimal efficacy and safety in those studies, providing a reliable foundation for our current research. After the final treatment dose, the rats underwent another MRI scan. Then, blood samples were collected by cardiac puncture under deep anesthesia with ketamine–xylazine combination (80 and 8 mg kg^−1^,

respectively). Following blood collection, brain tissues were carefully harvested by craniotomy and stored for subsequent analysis.

### Magnetic resonance imaging (MRI)

The animals were anesthetized intraperitoneally while lying supine with their hind limbs positioned parallel. Magnetic resonance imaging (MRI) was conducted using a 3 Tesla clinical scanner (Magnetom Skyra; Siemens Healthineers, Erlangen, Germany), with the rats positioned prone. T2-SPACE (sampling perfection with application-optimized contrast using different flip angle evolution) sequences were employed to visualize the brain in both coronal and transverse planes. The T2-SPACE sequence parameters included a TR (repetition time) of 6000 ms, TE (echo time) of 100 ms, voxel size of 0.5 × 0.5 × 1 mm, 34 slices with a slice thickness of 2 mm, and slice spacing of 0.6 mm. Manual measurements were conducted to assess tumor volume. A radiologist measured tumor length (L), width (W), and height (H), then computed the tumor volume using the formula: 0.5236 × L × W × H [32].

### Hematological and biochemical analyses

For hematological analysis, the blood samples were analyzed within 60 min of being collected. On the morning of the analysis, the automated hematology system (Archem, BM240, Istanbul, Turkey) was verified against a reference sample provided by the manufacturer, followed by a control sample analysis cycle. The measured parameters included white blood cell (WBC) count, red blood cell (RBC) count, hemoglobin (HGB) level, hematocrit (HCT) level, mean cell volume (MCV), mean corpuscular hemoglobin (MCH), mean corpuscular hemoglobin concentration (MCHC), and platelet (PLT) count.

For biochemical analysis, blood samples were maintained at 20 °C after collection and thorough mixing. They were then analyzed within 60 min of collection using the BS-200 Automatic Biochemistry Analyzer (Mindray Co., Shenzhen, P. R. China). Before starting the analysis, calibration was performed on the analyzer using calibration samples with known plasma levels for each parameter. Calibration occurred before analysis initiation, periodically during analysis of study samples, and after all analyses were completed. The measured parameters included alkaline phosphatase (ALP), alanine aminotransferase (ALT), aspartate aminotransferase (AST), lactate dehydrogenase (LDH), triglycerides (TG), total cholesterol (TCHOL), HDL- and LDL-Cholesterol (HDL-C and LDL-C), glucose (GLU), Creatinine (CREA), and uric acid (UA). For every study sample, the Animal ID and Sample number were manually inputted into the analyzer. The analyzer was configured for automatic print-out, and each individual sample print-out was gathered and stored in the study folder.

### Histopathological examinations

The brain tissue samples were first fixed in a 10% buffered formaldehyde solution. After fixation, they underwent a series of processing steps involving graded alcohol and xylene, ultimately being embedded in paraffin blocks. Subsequently, sections with a thickness of 5 µm were taken from these paraffin blocks at intervals of 50–100 µm. Hematoxylin and eosin staining was then applied to these sections to assess histopathological alterations. The histopathological evaluations included assessing the size of the tumor focus, necrotic area, vascular proliferation, and cellular proliferation patterns. The size of the tumor focus was categorized based on the diameter measured using a 4X objective on the microscope: > 3500 µm was classified as very severe, 3499–2000 µm as severe, 1999–1000 µm as moderate, 999–500 µm as mild, and < 500 µm as very mild. Similarly, the size of the necrotic area was classified based on its diameter: > 1500 µm as very severe, 1499–1000 µm as severe, 999–500 µm as moderate, 499–200 µm as mild, and < 199 µm as very mild.

### Immunohistochemical examinations

For immunohistochemical examinations, primary antibodies were used, including Ki67 (Cat no: ab15580, Dilution ratio: 1/100), CMPK2 (Cat no: PA5-34461, Dilution ratio: 1/100), and glial fibrillary acid protein (GFAP) (Cat no: ab6842, Dilution ratio: 1/100). The chromogen employed was 3-3′ diaminobenzidine (DAB), with Mayer’s hematoxylin utilized for counterstaining. The sections were examined under a light microscope (Zeiss AXIO, Germany).

### Double immunofluorescence examination

In immunofluorescence examinations, the tissue samples were first treated with the primary antibody NOP10 (Cat no: ab134902, Dilution ratio: 1/100) and incubated according to the provided instructions. Then, a fluorescent secondary antibody (FITC, Cat No: ab6785, Dilution Ratio: 1/1000 UK) was applied and left to incubate in the dark for 45 min. Subsequently, a second primary antibody (H2A.X, Cat no: sc-517336, Dilution ratio: 1/100) was applied and incubated following the instructions. Next, a different fluorescent secondary antibody (Texas Red, Cat No: ab6719, Dilution Ratio: 1/1000 UK) was applied and incubated in the dark for 45 min. Finally, DAPI (Cat no: D1306, Dilution Ratio: 1/200 UK) was applied to the sections for 5 min in the dark, after which the tissues were covered with coverslips. The stained tissues were then examined under a fluorescence attachment-equipped microscope (Zeiss AXIO, Germany).

### Genotoxicity assessment

For the peripheral blood micronucleus (MN) assay, blood smears were prepared on clean microscope slides, left to air-dry, fixed with methanol, and subsequently stained with Giemsa (10% in 0.1 mM sodium phosphate buffer, pH 6.8) for 10 min. Following rinsing with PBS, the slides were examined under a light microscope. The frequencies of micronuclei (MN) were determined by assessing 1000 cells per animal. Representative photomicrographs were taken to document the presence of micronuclei [35].

### Statistical analysis

The results are presented as means ± S.D. and were evaluated for statistical significance using one-way analysis of variance (ANOVA), followed by post hoc analysis for multiple comparisons using Duncan’s test. To assess the density of positive staining in both immunohistochemical and immunofluorescence staining images, five randomly chosen areas were analyzed using ZEISS Zen Imaging Software. The data were statistically characterized as mean and standard deviation (mean ± SD) for the percentage of the area. The statistical analysis was performed using the Instat software package (GraphPad Software, San Diego, CA, USA).

## Results

**Fig. 1:**
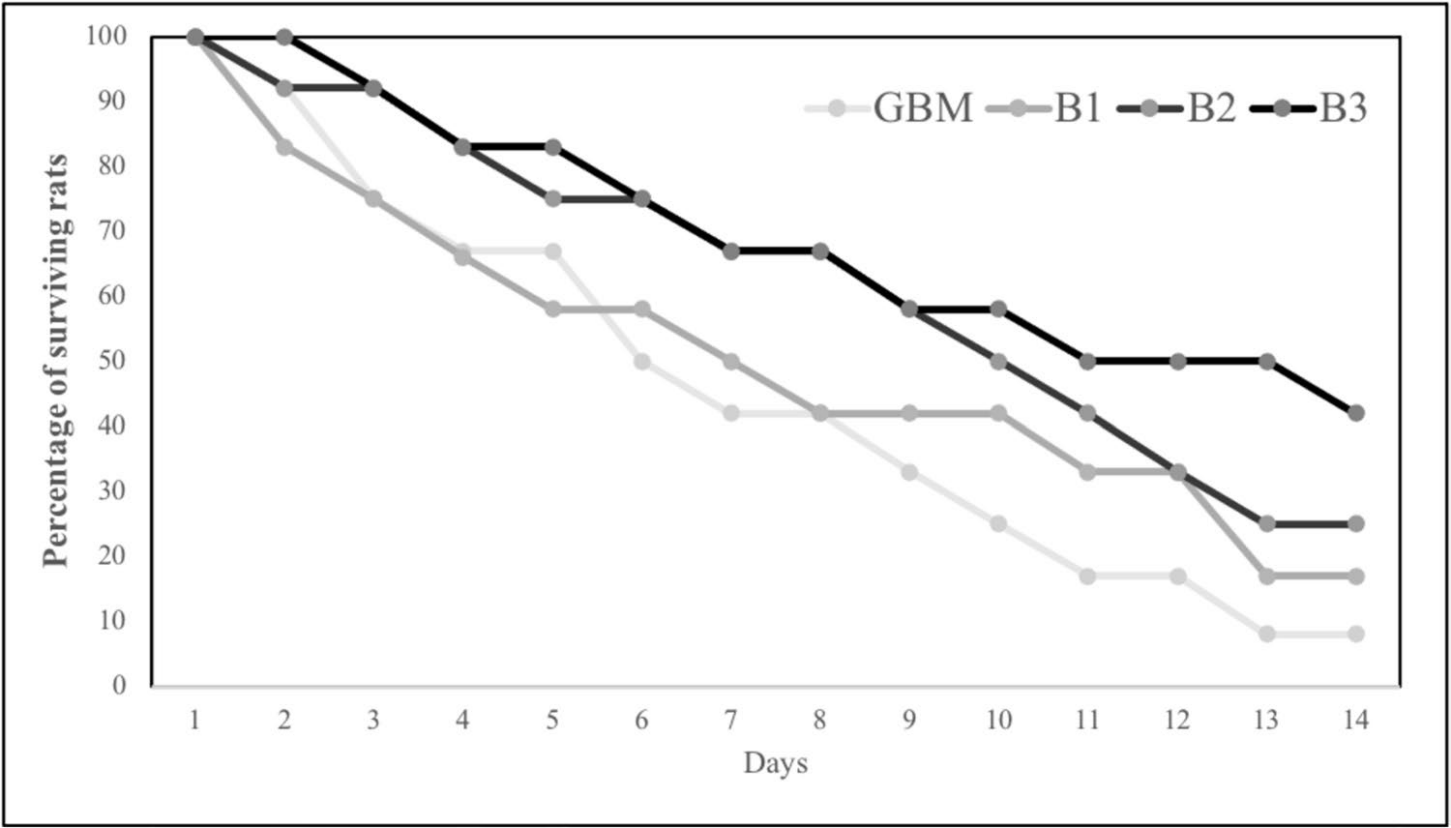
The survival rates of rats within each experimental group

Overall survival was measured from the initiation of tumor induction (14th day) until either death or the conclusion of the experiment (28th day). All doses of BA extended survival compared to control rats (GBM, 14 days). Animals in B3 group exhibited superior survival outcomes compared to animals in B1 and B2 groups (Fig. 1). On the other hand, the GBM group showed a reduction in body weight compared to the healthy control group. However, no significant increase in body weight was noted in the all BA treatment groups when compared to the GBM group.

**Fig. 2:**
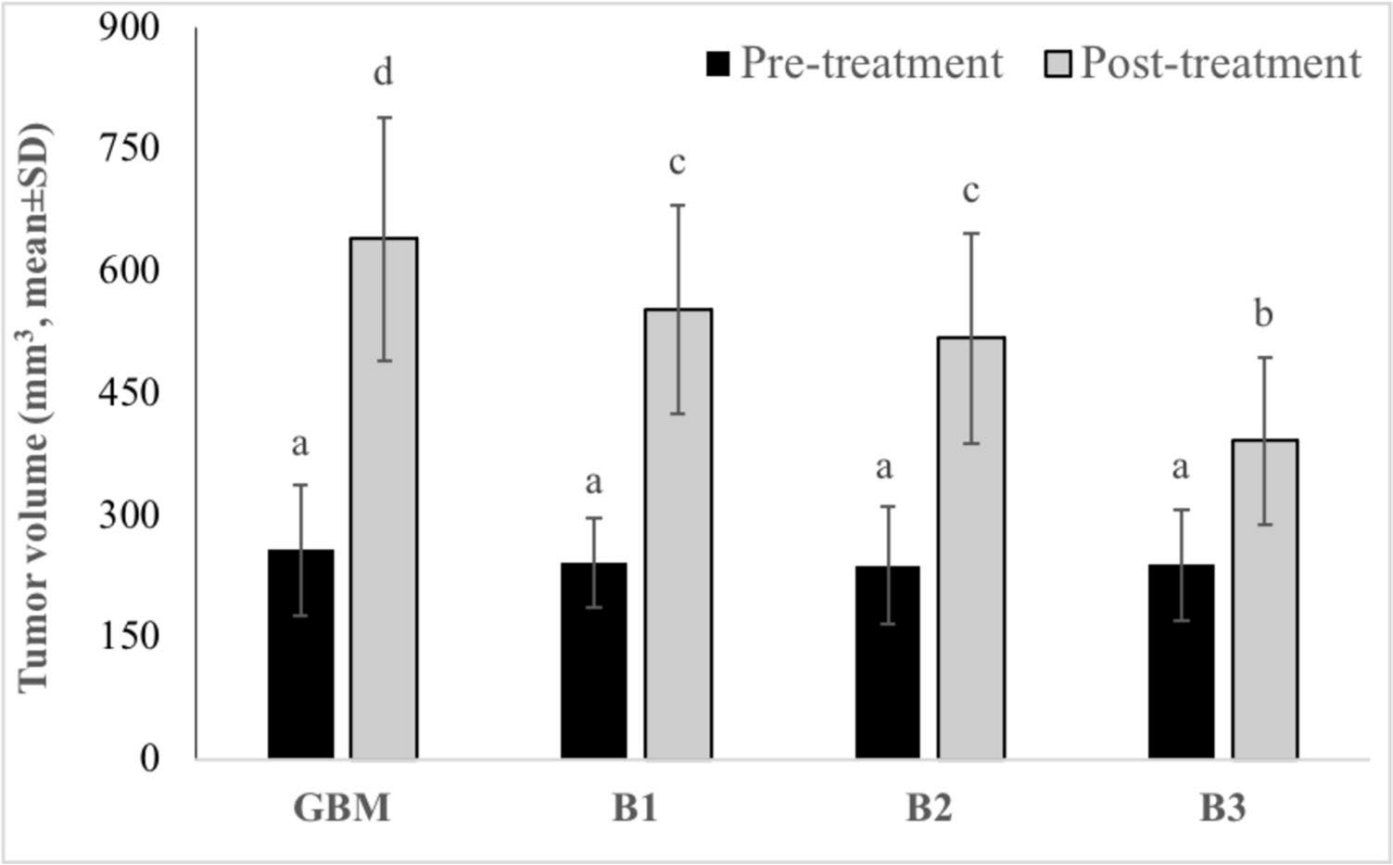
MRI monitoring of tumor advancement in the GBM model after treatment with different BA doses. Different letters indicate statistically significant differences (*p* < 0.05)

**Fig. 3:**
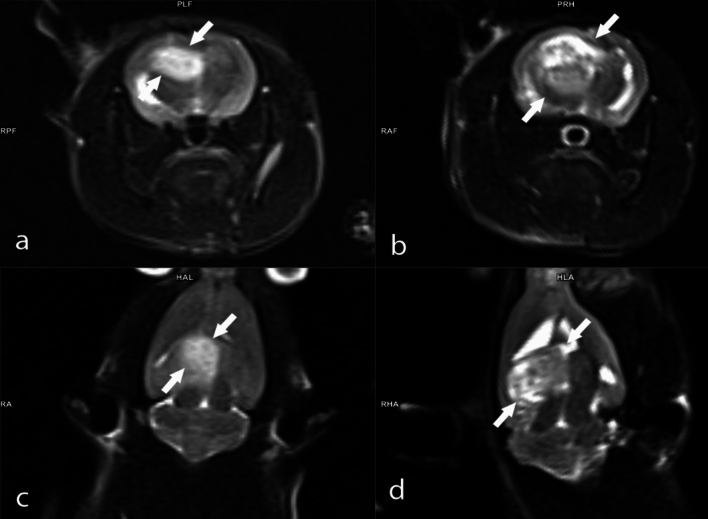
MRI images of the rat brain with GBM: **A** axial T2-weighted MRI image taken on the 14th day post-implantation; **B** axial T2-weighted MRI image captured on the 28th day post-implantation; **C** coronal T2-weighted MRI image obtained on the 14th day post-implantation; **D** coronal T2-weighted MRI image acquired on the 28th day post-implantation

**Fig. 4:**
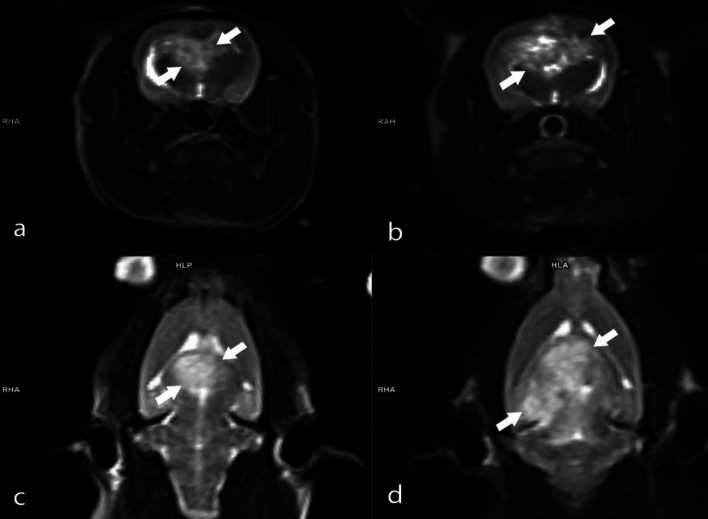
MRI images of the rat brain with GBM: **A** Axial T2-weighted MRI image obtained on the 14th day post-implantation; **B** Axial T2-weighted MRI image following treatment with B1 on the 28th day; **C** Coronal T2-weighted MRI image taken on the 14th day post-implantation; **D** Coronal T2-weighted MRI image after treatment with B1 on the 28th day

**Fig. 5:**
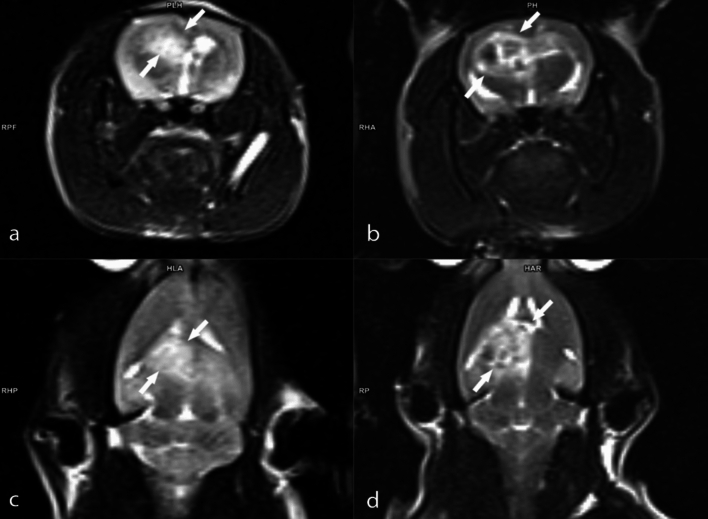
MRI images of the rat brain with GBM: **A** Axial T2-weighted MRI image obtained on the 14th day post-implantation; **B** Axial T2-weighted MRI image following treatment with B2 on the 28th day; **C** Coronal T2-weighted MRI image taken on the 14th day post-implantation; **D** Coronal T2-weighted MRI image after treatment with B2 on the 28th day

**Fig. 6:**
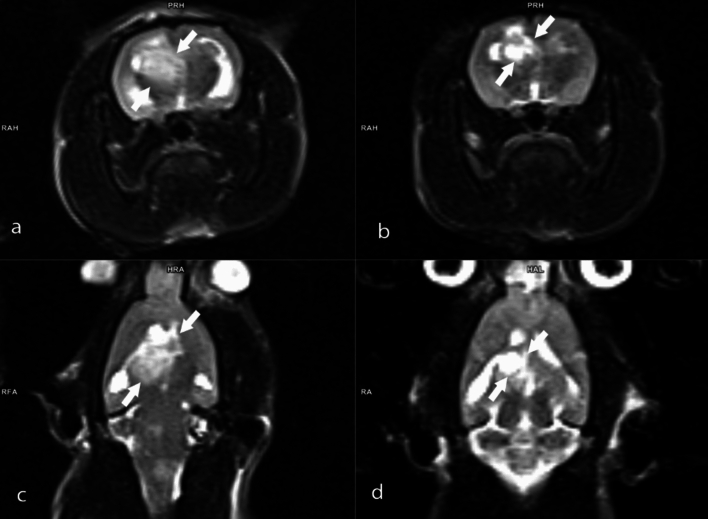
MRI images of the rat brain with GBM: **A** axial T2-weighted MRI image obtained on the 14th day post-implantation; **B** Axial T2-weighted MRI image following treatment with B2 on the 28th day; **C** Coronal T2-weighted MRI image taken on the 14th day post-implantation; **D** Coronal T2-weighted MRI image after treatment with B2 on the 28th day

Tumor volumes were assessed using MRI 14 days after tumor implantation, with animals having a tumor volume of 50 mm^3^ and above being included in the experiments. Three different doses of BA (3.25, 6.5, and 13 mg kg^−1^ b.w) were administered to animals with confirmed tumor formation. The percentages of tumor size elevations (as mm^3^) were determined as 248.1%, %227.9%, 216.7%, and 163.3% for the experimental groups including GBM model, B1, B2, and B3, respectively. The antitumor activities of the tested BA doses were ranked as follows: B3 > B2 > B1 (Fig. 2). Considering the tumor sizes obtained from MRI analyses, it was found that the anti-GBM effect of BA was clearly dose dependent (Figs. 3, 4, 5, 6).

Hematology and biochemistry data were reflected in Tables 1 and 2, respectively. Hematological values measured showed a significant (*p* < 0.05) decrease of WBC level in GBM group. Furthermore, significant elevations in WBC were recorded for applications with BA after the 14-day treatment. Conversely, the PLT count was significantly elevated in GBM group compared to the control group. Treatments with all doses of BA resulted in reductions in PLT counts compared to the GBM group, although the measured PLT counts remained higher than those of the healthy control group. Other hematological parameters including red blood cell (RBC) count, hemoglobin (HGB) level, hematocrit (HCT), mean cell volume (MCV), mean corpuscular hemoglobin (MCH), and mean corpuscular hemoglobin concentration (MCHC) did not show significant differences in all treatments compared to the control rats, and they remained within the normal range (Table 1).

**Table 1:** Effect of boron treatments on hematological parameters of control and experimental groups [untreated (GBM group); treatment with 3.25 mg kg^−1^ BA (B1 group); treatment with 6.5 mg kg^−1^ BA (B2 group); treatment with 13 mg kg^−1^ BA (B3 group)]

Groups	WBC (10^9^ L^−1^)	RBC (10^12^ L^−1^)	HGB (g dL^−1^)	HCT (%)	MCV (fL)	MCH (pg)	MCHC (g dL^−1^)	PLT (10^9^ L^−1^)
Control	4.9 ± 1.1	7.2 ± 1.4	15.3 ± 2.4	48.4 ± 3.9	58.6 ± 6.3	19.5 ± 3.5	31.4 ± 4.1	612.2 ± 57.2
GBM Model	2,1 ± 0.9*	7.9 ± 1.1	16.2 ± 2.3	50.1 ± 4.6	57.2 ± 6.6	17.8 ± 2.9	31.1 ± 2.9	924.6 ± 148.1*
B1 (3.25 mg kg^−1^ BA)	4.6 ± 1.3	7.4 ± 1.6	15.7 ± 2.1	47.4 ± 5.3	57.6 ± 5.0	18.0 ± 2.8	30.5 ± 3.8	782 ± 95.3*
B2 (6.5 mg kg^−1^ BA)	4.8 ± 0.9	7.6 ± 1.2	15.4 ± 1.8	48.5 ± 3.8	57.7 ± 5.5	18.1 ± 3.0	30.3 ± 4.0	845 ± 102.4*
B3 (13 mg kg^−1^ BA)	4.3 ± 1.0	7.5 ± 0.8	15.3 ± 2.2	46.9 ± 5.7	59.4 ± 6.1	17.9 ± 2.6	30.5 ± 3.7	866 ± 91.5*

*Symbol represents significant alterations at *p* < 0.05 in comparison to control values. Values are expressed as mean ± standard deviation. *WBC* white blood cells, *RBC* red blood cells, *HGB* hemoglobin, *HCT* hematocrit, *MCV* mean cell volume, *MCH* mean cell hemoglobin, *MCHC* mean cell hemoglobin concentration, *PLT* platelet)

**Table 2:** Effect of boron treatments on hematological parameters of control and experimental groups [untreated (GBM group); treatment with 3.25 mg kg^−1^ BA (B1 group); treatment with 6.5 mg kg^−1^ BA (B2 group); treatment with 13 mg kg^−1^ BA (B3 group)]

Groups	ALP (IU L^−1^)	AST (IU L^−1^)	ALT (IU L^−1^)	LDH (IU/L)	TG (mmol L^−1^)	TCHOL (mmol L^−1^)	HDL-C (mmol L^−1^)	LDL-C mmol L^−1^	GLU mmol L^−1^	CREA mmol L^−1^	UA mmol L^−1^
Control	114.6 ± 11.3	93.5 ± 8.3	34.8 ± 5.9	456.7 ± 48.1	114.5 ± 9.7	63.4 ± 5.3	46.9 ± 3.1	5.9 ± 1.0	285.4 ± 21.2	0.37 ± 0.1	0.64 ± 0.1
GBM Model	88.3 ± 10.1*	172.4 ± 13.6*	76.9 ± 6.5*	714.4 ± 52.6*	62.1 ± 7.0*	60.1 ± 5.8	44.2 ± 3.3	8.3 ± 1.5*	299.2 ± 26.6	0.44 ± 0.1*	3.84 ± 0.6*
B1 (3.25 mg kg^−1^ BA)	109.5 ± 9.8	152.2 ± 11.8*	74.4 ± 5.2*	581.0 ± 38.8*	85.5 ± 9.6*	59.2 ± 6.1	40.8 ± 4.6	5.4 ± 1.4	314.3 ± 27.0	0.35 ± 0.1	1.52 ± 0.3*
B2 (6.5 mg kg^−1^ BA)	113.2 ± 10.7	161.1 ± 13.4*	69.8 ± 6.1*	602.3 ± 44.5*	91.3 ± 10.2*	58.8 ± 4.9	42.5 ± 3.9	6.6 ± 1.3	318.6 ± 19.8	0.38 ± 0.1	1.41 ± 0.4*
B3 (13 mg kg^−1^ BA)	120.8 ± 14.1	164.6 ± 12.0*	75.5 ± 6.3*	566.7 ± 63.5*	66.7 ± 7.9*	57.1 ± 6.7	41.3 ± 4.4	6.1 ± 1.4	321.2 ± 25.7	0.38 ± 0.1	1.89 ± 0.7*

*Represents significant alterations at *p* < 0.05 in comparison to control values. Values are expressed as mean ± standard deviation. *ALP* alkaline phosphatase, *ALT* Alanine aminotransferase, *AST* aspartate aminotransferase, *LDH* lactate dehydrogenase, *TG* triglyceride, *TCHOL* total cholesterol, *HDL-C* high-density lipoprotein cholesterol, *LDL-C* low-density lipoprotein cholesterol, *GLU* glucose, *CREA* creatinine, *UA* uric acid)

The effects of treatments with BA on biochemical parameters are illustrated in Table 2. Levels of AST, ALT, LDH, LDL-C, CREA, and UA were significantly elevated in rats with GBM compared to control rats. The enhanced levels of LDL-C and CREA in the GBM group were significantly reduced (*p* < 0.05) by all BA applications. Similarly, levels of ALP and TG were significantly decreased in rats with GBM compared to control rats. The reduced levels of ALP in the GBM group were significantly elevated (*p* < 0.05) by all BA applications. Other biochemical parameters measured, including TCHOL, HDL-C, and GLU in the treatment rats, did not exhibit significant differences compared to the control group (Table 2).

**Fig. 7:**
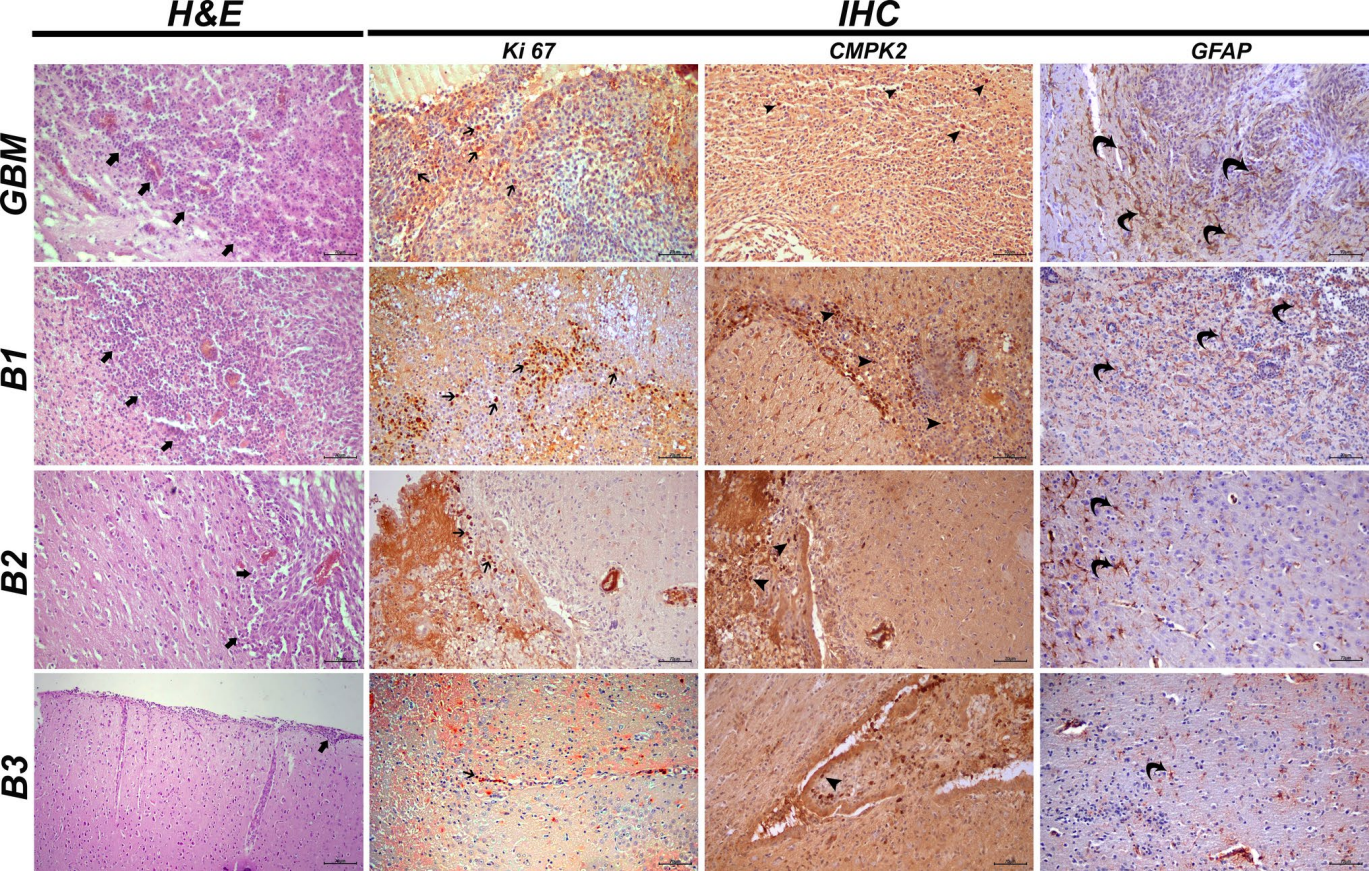
Brain tissue, glioblastoma, tumor foci (thick arrows), H&E staining, scale bar: 70 µm. Ki67 expressions in the tumor cell (thin arrows), CMPK2 expressions in anaplastic cells (arrow heads), and GFAP expressions in astrocytes surrounding the mass (curved arrows) detected via IHC-P, Scale bar: 40 µm

**Fig. 8:**
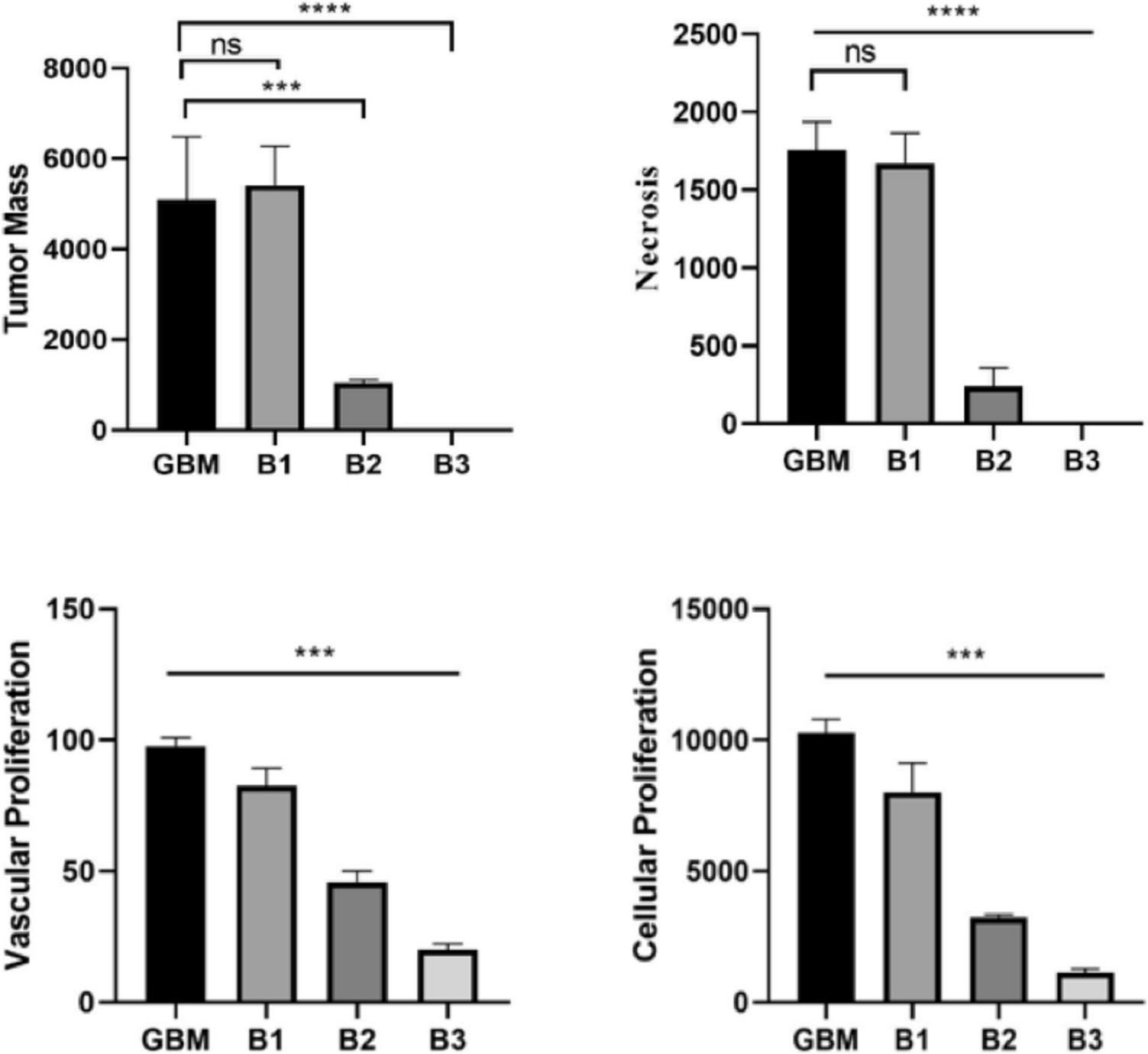
Histopathological findings observed in brain tissues: tumor mass: (****p < 0.0001, ***p = 0.0007, ns *p* = 0.3550); Focus of Necrosis: (****p < 0.0001, ns *p* = 0.9136); Vascular proliferation: (***p = 0.0003, *** *p* = 0.0002 (GBM vs B3); Cellular proliferation: (***p = 0.0007, ***p = 0.0002 (GBM vs B3)

In the GBM group, wide tumor foci containing the gray and white matter layers of the brain tissues were observed in the histopathological examination. Widespread necrotic foci were present in the central regions of the tumor foci, while in the areas close to healthy tissue, there was a very severe level of vascular proliferation. Particularly in the vicinity of the vessels, anaplastic cells exhibited a very severe level of mitotic figures and hyperchromasia. Additionally, severe hyperemia was identified in the vessels in this area (Fig. 7). In histopathological evaluation of brain tissues of B1 group, we observed widespread tumor foci spanning both the gray and white matter layers. These foci displayed extensive necrotic regions and exhibited a significantly elevated level of vascular and cellular proliferation, primarily within their central regions. Notably, we observed a severe presence of mitotic figures and intense hyperchromasia in cells, particularly in areas adjacent to healthy tissue and surrounding blood vessels. Additionally, severe hyperemia was evident in the blood vessels surrounding the tumor mass (Fig. 7). Upon histopathological examination of brain tissues in B2 group, we noted a reduction in the diameter of the tumor focus compared to the GBM group. The mass demonstrated mild vascular and cellular proliferation, with few mitotic cells observed at its periphery. Furthermore, we detected moderate hyperemia in the blood vessels within the healthy tissues surrounding the tumor mass (Fig. 7). Our histopathological examination of brain tissues in B3 group revealed only a small number of anaplastic cells surrounding blood vessels in the gray matter layer (Fig. 7). A summary of the histopathological findings was presented in Fig. 8.

**Fig. 9:**
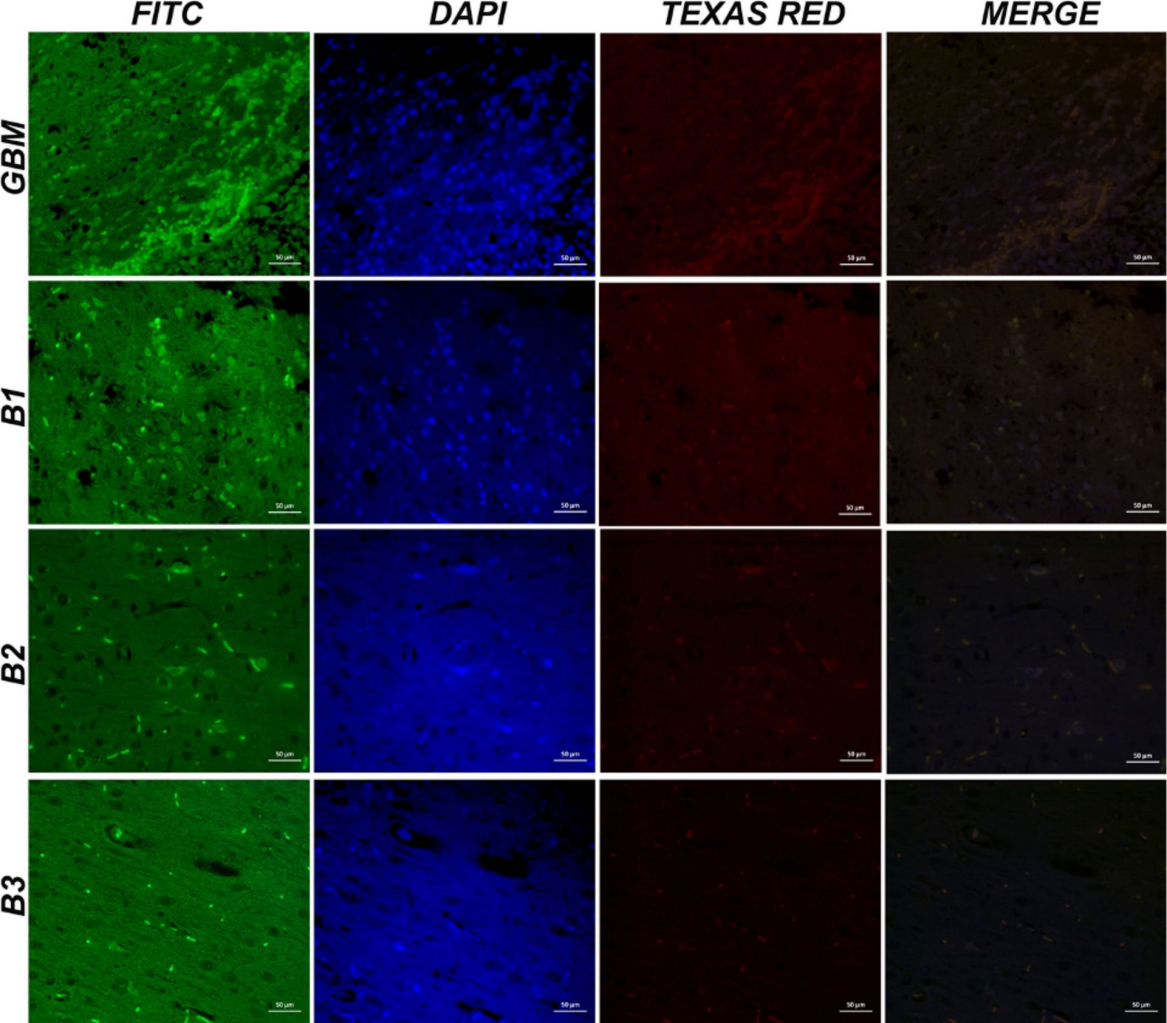
Brain tissues with GBM showing NOP10 and H2A.X expressions in neurons, immunofluorescence staining. Scale bar: 50 µm

**Fig. 10:**
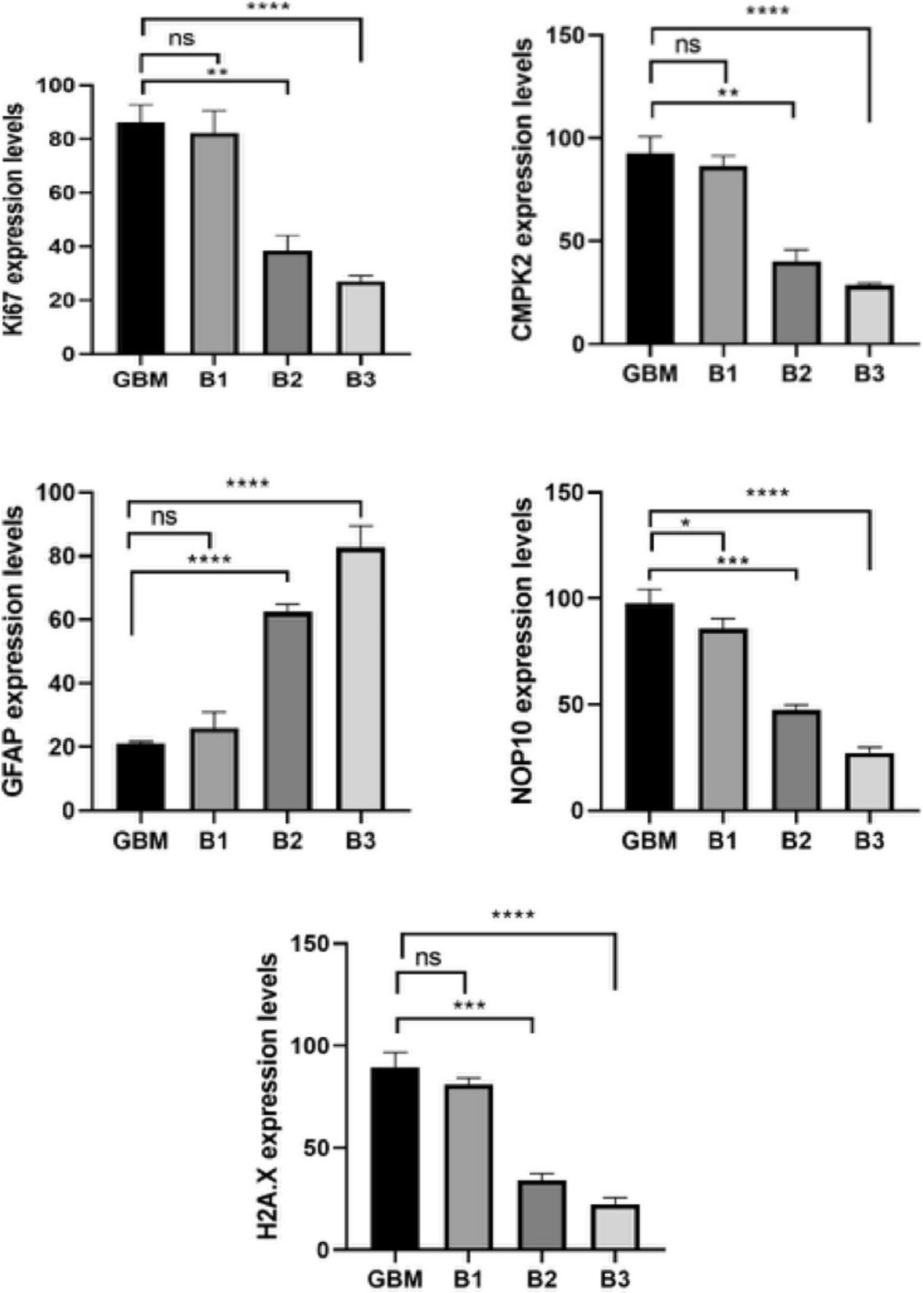
Immunohistochemical and immunofluorescence staining data. Ki67: (****p < 0.0001, **p = 0.0010, ns *p* = 0.8510); CMPK2: (****p < 0.0001, **p = 0.0012, ns *p* = 0.4314); GFAP: (****p < 0.0001, ns *p* = 0.2238); NOP10: (****p < 0.0001, ***p = 0.0002, *p = 0.0480); H2A.X: (****p < 0.0001, ***p = 0.0003, ns *p* = 0.0979); ns indicates no significant difference

In the immunohistochemical and immunofluorescence staining of brain tissues of GBM group, we observed severe intracytoplasmic Ki67 expressions around blood vessels and in cells adjacent to healthy tissue. Additionally, severe cytoplasmic CMPK2 expressions were observed in anaplastic cells, while mild GFAP expressions were noted in astrocytes surrounding the mass (Fig. 7). In the immunofluorescence examination, severe intracytoplasmic Nop10 and H2AX expressions were detected around the tumor mass (Fig. 9). Immunohistochemical and immunofluorescence staining of brain tissues of B1 group revealed severe cytoplasmic Ki67 expressions in tumor cells around blood vessels and in areas near healthy tissue. Moreover, severe intracytoplasmic CMPK2 expressions were observed in anaplastic cells, along with mild GFAP expressions in astrocytes surrounding the mass (Fig. 7). In the immunofluorescence examination, severe intracytoplasmic Nop10 and H2AX expressions were found in neurons around the tumor mass (Fig. 9). Besides, moderate intracytoplasmic Ki67 and CMPK2 expressions were detected in tumor cells around blood vessels in B2 group. Concurrently, severe GFAP expression was observed in astrocytes surrounding the tumor (Fig. 7). In the immunofluorescence examination, moderate Nop10 and H2AX expressions were identified in neurons around the tumor mass in B2 group (Fig. 9). Again, B3 revealed mild cytoplasmic Ki67 and CMPK2 expressions in tumor cells around blood vessels, along with moderate GFAP expression in astrocytes surrounding the tumor (Fig. 7). Additionally, mild intracytoplasmic Nop10 and H2AX expressions were observed in neurons around the tumor mass in brain tissues (Fig. 9). Data and statistical analysis findings for immunohistochemical and immunofluorescence staining are summarized in Fig. 10.

The results from the MN test, as presented in Table 3, demonstrate that the GBM group exhibited a significantly higher frequency of micronucleated erythrocytes compared to the healthy group (*p* < 0.05). 3.25 mg kg^−1^, 6.5 mg kg^−1^, and 13 mg kg^−1^ doses of BA treatments (B1, B2, and B3 groups) resulted in slight reductions in the number of micronuclei formations compared to the GBM group.

**Table 3:** The assessment of micronuclei rates in the peripheral blood of rats subjected to an experimental model of GBM [untreated (GBM group); treatment with 3.25 mg kg^−1^ BA(B1 group); treatment with 6.5 mg kg^−1^ BA (B2 group); treatment with 13 mg kg^−1^ BA (B3 group)]

Groups	MN frequency
Control	2.34 ± 0.63^a^
GBM model	5.66 ± 0.79^b^
B1 (3.25 mg kg^−1^ BA)	5.30 ± 1.11^b^
B2 (6.5 mg kg^−1^ BA)	5.16 ± 1.24^b^
B3 (13 mg kg^−1^ BA)	5.04 ± 1.08^b^

A comprehensive analysis of 1000 cells per sample was conducted. Results are presented as mean ± standard deviation. Different letters indicate statistically significant differences (*p* < 0.05)

## Discussion

Boron-based compounds have gained significant attention as potential therapeutic agents for glioblastoma multiforme (GBM) due to their unique mechanisms of action and promising preclinical safety profiles [36]. However, challenges such as bioavailability and blood–brain barrier (BBB) penetration must be addressed before their clinical implementation. Strategies like boron-carrier nanoparticles, liposomal formulations, and receptor-targeted delivery systems are currently being explored to enhance boron delivery across the BBB [37–40]. Although boron-based compounds have demonstrated substantial antitumoral effects in preclinical models, limited in vivo and clinical data exist, and further research is required to assess their efficacy, pharmacokinetics, and safety in clinically relevant settings [20, 41]. Long-term studies are essential to establish their therapeutic potential and identify any potential off-target effects and stability under physiological conditions. When compared to other therapies for GBM, such as targeted molecular inhibitors, immunotherapies, and nanoparticle-based approaches, boron-based compounds offer distinct advantages. These include the potential to target multiple cellular pathways simultaneously, thereby reducing the likelihood of resistance [42]. Boron-based agents could also complement immunotherapy strategies by enhancing immune activation and directly targeting tumor cells. Nanoparticles, especially boron-containing ones, have shown promise in optimizing drug delivery for therapies like boron neutron capture therapy (BNCT), thus improving therapeutic outcomes [20, 42, 43].

This study rigorously examined the anticancer efficacy of B supplementation (in the form of BA) in a glioblastoma (GBM) xenograft animal model, employing a comprehensive, multifaceted approach. Tumor progression was quantitatively analyzed using MRI, alongside evaluations of survival rates, hematological and biochemical parameters. Detailed histopathological, immunohistochemical, and immunofluorescence analyses were also conducted. The findings indicate that BA impedes GBM progression, enhancing rat survival by regulating Ki67 expression, as well as cytoplasmic CMPK2 and GFAP levels within tumors. This study unveils, for the first time, the pharmacological promise of BA in addressing GBM, offering valuable insights for GBM therapeutic strategies. BCCs, especially BA and BX, exerted anticancer effects in hepatocellular carcinoma [44], endometrial cancer [45], small-cell lung cancer [46], prostate cancer [47], breast cancer [48], and GBM [20]. Hence, B has emerged as a vital tool in anticancer research, attributed to its substantial impact on cell proliferation intervention [19]. In fact, recent studies have suggested that the application of B (as BA and BX) holds promise as an effective approach for treating GBM [49] and ferroptosis induction as well as the inhibition of cell proliferation was associated with the anti-GBM action [50]. However, there exists a gap in the literature regarding the exploration of the underlying mechanisms of anti-GBM action by BCCs.

GFAP is frequently used to visualize astrocytes and tumors originating from glial cells. Earlier studies investigating GFAP staining in high-grade glioma cells have revealed diminished GFAP expression in giant cell glioma. Likewise, a gradual decline in GFAP production was reported as the malignancy of astrocytoma cells increases [51]. The correlation between GFAP and overall survival remained consistent with the loss of GFAP in high-grade tumors and the subsequent increase in tumor growth [52]. In accordance with these findings, we observed mild GFAP expressions in astrocytes surrounding the tumor mass. Findings from injury models in experimental animals have indicated an enhanced fragility of the central nervous system (CNS) when GFAP is not present [53]. However, the molecular mechanism underlying the elevated expression of GFAP in astrocytes remains unclear, and studies employing in vitro models to explore GFAP's impact on cell proliferation yield inconclusive results [54], previous evidence has suggested that GFAP expression profoundly influenced cell morphology and suppressed cell growth in C6 cells, implying a potential tumor-suppressing role for GFAP in astrocytoma [55]. GFAP was discovered to regulate the multiple endocrine neoplasia type 1 (MEN1) tumor suppressor gene during the S and early G2 phases of the cell cycle through its interaction with MEN1 via its head domains [54, 56]. In this regard, our findings revealed that the supplementation with B led to remarkable elevation of GFAP expressions (from mild to moderate or severe amounts) in rodent GBM model. Thus, it can be suggested that targeting tumor suppressor networks with BCCs might hold tremendous therapeutic potential for the treatment of GBM.

The findings presented in the current study also demonstrate a dose-dependent reduction in Ki67, Nop10, and H2AX expressions in tumor cells around blood vessels, alongside GFAP expression, following treatment with BA in astrocytes surrounding the tumors in rat GBM model. The Ki67 labeling index is recognized as a significant indicator of tumor cell proliferation in cases of GBM, with high Ki67 levels being linked to poorer overall survival in patients with lower grade gliomas or ependymomas [57, 58]. Consistent with the current findings, several BCCs have been shown to reduce cell proliferation in melanoma, colon, prostate, and breast cancer cell lines [59]. Again, Nop10 protein expression was predominantly detected in the nucleus and nucleolus of invasive tumor cells, demonstrating a notable association with aggressive characteristics. Additionally, TP53 mutations were highly common in tumors displaying high NOP10 expression [60]. BCCs could disrupt the physiology and reproduction of cancer cells by inhibiting serine proteases and it was suggested that serine protease inhibitors might suppress metastasis, invasion as well as angiogenesis in several cancers [61]. H2AX also functions as a marker for identifying apoptotic suppression status, genomic instability, metastatic progression, and the survival capability of GBM cells [62]. BCCs such as tungsten boride and boron carbide have been documented to positively impact angiogenesis, cytokine and chemokine metabolism, and impede migration or invasion by activating various genes. Additionally, they have been found to modulate the TGF-beta signaling pathway, resulting in the inhibition of malignant cell growth in human alveolar epithelial cells [63, 64]. The majority of data from preclinical studies indicated that BA played a pivotal role as a tumor suppressor [45]. Hence, the observed anti-GBM action by BA could be attributed, to a greater or lesser extent, to its tumor-suppressing potency associated with GFAP, Ki67, Nop10, and H2AX expressions.

CMPK2 has recently emerged as a promising novel therapeutic target for the development of new drugs to treat GBM. Our immunohistochemical analysis also revealed significant cytoplasmic expression of CMPK2 in anaplastic cells in rat GBM model. CMPK2, a key gene involved in DNA synthesis, plays an essential role in cell growth and is found to be elevated in tumor cells while not in normal brain cells [65]. Indeed, the overexpression of CMPK2 has been found to disrupt normal mitochondrial physiology, as indicated by elevated reactive oxygen species (ROS) amounts and decreased membrane potential. As a result of this disruption, the increased activation of the extracellular-signal-regulated kinase (ERK) signaling and a remarkable elevation in the production of proinflammatory mediators such as TNFα, IL8, and IL1β occurred [66]. Pro-inflammatory mediators and proliferative microglia have been identified as key contributors to the progression of GBM [67]. Indeed, the NLRP3 inflammasome was found to contribute to therapy resistance in U87 and GL261 xenograft mouse GBM models. Inhibition of this inflammasome increased animal survival and decreased tumor growth following GBM treatment [68]. Our results indicated a decrease in CMPK2 expression in tumor cells surrounding blood vessels, especially in animals administered 13 mg/kg of BA. The anti-GBM action of BA associated with CMPK2 inhibition could be attributed to the alleviation of cellular injuries such as resulting from inflammatory changes [20]. Supporting our findings, interestingly, evidence suggested that BCCs might exert beneficial action by influencing immune-related genes such as protein kinase A (PKA) and ERK through the MAPK signaling pathway [69]. Furthermore, novel boron-based inhibitors of the NLRP3 inflammasome, such as 2-aminoethoxy diphenylborinate, have recently been introduced for inhibiting the NLRP3 inflammasome both in vitro and in vivo, without affecting Ca^2+^ homeostasis [70]. Collectively, our findings suggest that B indeed holds promise as a CMPK2 inhibitor for the treatment of GBM.

In our study, we not only assessed the in vivo anti-GBM activity of B but also conducted genotoxicity assessment along with hematology and preclinical biochemistry analyses. This comprehensive approach aimed to evaluate the biosafety of B applications at the preclinical level.

Our study yielded a result consistent with existing literature, indicating that B or BCCs compounds exhibit low genotoxicity potential or are non-genotoxic [9, 17, 18, 71]. There was no observed increase in MN frequencies in animals supplemented with B compared to the GBM group. The hematological analysis revealed a remarkable (*p* < 0.05) reduction in WBC count within the GBM group. In line with our findings, it was noted a prominent rise in WBC levels among glioma patients in comparison to patients with meningioma, neuralgia, and neuroma. Although WBC count is not currently recognized as a blood marker for GBM, increased neutrophil counts have long been associated with tumor advancement [72]. Indeed, the correlation between the levels of various cytokines in GBM cyst fluid and WBC counts executed that cystic GBMs created highly inflammatory environments. These environments interacted with the circulation and caused both displacement and destruction of brain tissue [73]. Our findings indicated that the PLT count exhibited a significant increase in the GBM group when compared to the control group. The precise interaction between PLTs and the growth of GBM remains uncertain. It has been previously suggested that growth factors released by PLTs could facilitate the growth and migration of malignant glioma and endothelial cells, potentially contributing to enhanced angiogenesis within tumors [74]. Conversely, significant increases in WBC levels were observed after 14 days of treatment with B applications, and treatment with all doses of B applications led to slight reductions in PLT counts compared to the GBM group. The positive changes observed in WBC and PLT counts in this study could be attributed to the anti-inflammatory [20, 75] and antimigration [76, 77] properties of B or BCCs. The carried out preclinical biochemistry analysis proved that the reduced levels of ALP in the GBM group were significantly elevated by all B supplementations. Similarly, the elevated levels of LDL-C and CREA in the GBM group were significantly decreased (*p* < 0.05) by all B applications. Increased levels of ALPs in the blood have been associated with metastases in various cancers such as prostate and breast cancers. Interestingly, studies have shown that inhibitors targeting ALPs can reduce the growth and invasion of tumor cells. This suggests that ALP inhibitors could potentially be explored as a therapeutic approach to mitigate cancer progression and metastasis [78]. The observed positive biochemical changes suggest the presence of antimetastatic, nephroprotective, and lipid regulatory roles that develop following supplementation with B. Our findings align with the previous clinical and preclinical studies, further supporting the potential therapeutic benefits of B supplementation in various conditions [18, 79–81].

In conclusion, the antitumoral effects of boron in GBM have been thoroughly elucidated in this current study. We proposed primarily that targeting tumor suppressor networks with boron-containing compounds could offer considerable therapeutic promise for addressing GBM. The potential mechanisms underlying this antitumoral action may be associated with several factors, including the elevation of GFAP expressions, reduction of tumor cell proliferation, targeting tumor suppressor networks, suppression of metastasis, impeding migration or invasion, and influencing immune-related pathways. Our findings also revealed that boron indeed showed promise as a CMPK2 (an innovative drug target) inhibitor for the treatment of GBM. The hematological, biochemical, and genotoxicity assessments underscored the biosafety of boron applications in the treatment of GBM. Despite these promising preclinical findings, the study is inherently limited by its animal model context, and extrapolation to human GBM requires caution. Further research is essential to delineate the precise molecular pathways involved and to evaluate the long-term safety and efficacy of BA in clinical settings. This study lays the groundwork for future investigations, underscoring BA’s potential as a therapeutic agent for GBM and contributing valuable insights into its mechanistic actions.

## Data Availability

No datasets were generated or analyzed during the current study.
